# Efficient Colorimetric Fluoride Anion Sensor Based on π-Conjugated Carbazole Small Molecule

**DOI:** 10.3389/fchem.2021.732935

**Published:** 2021-08-25

**Authors:** Zhifeng Deng, Cheng Wang, Junqiang Li, Meng Zheng

**Affiliations:** ^1^ National and Local Joint Engineering Laboratory for Slag Comprehensive Utilization and Environmental Technology, School of Materials Science and Engineering, Shaanxi University of Technology (SNUT), Hanzhong, China; ^2^ Key Laboratory of Rubber–Plastic of Ministry of Education (QUST), School of Polymer Science and Engineering, Qingdao University of Science and Technology, Qingdao, China; ^3^ Qingdao Haiwan Science and Technology Industry Research Institute Co. Ltd., Qingdao, China

**Keywords:** fluoride anion sensors, naked eye detection, carbazole, intermolecular proton transfer, color change

## Abstract

The ability to detect fluoride anions with high selectivity and sensitivity by using the naked eye is crucial yet challenging. In this study, a novel, simple conjugated organic dye, *N*-*tert*-butyldimethylsilyl-3,6-diiodocarbazole (CA-TBMDS) was developed and used for the first time as a colorimetric sensor for fluoride. CA-TBMDS was found to be a highly sensitive fluoride chemosensor, with a detection limit as low as 3 × 10^−5^ M. The reaction of CA-TBMDS with fluoride anions in a tetrahydrofuran solution resulted in a color change from colorless to yellow under ambient light, which can be discriminated by the naked eye. The sensor operated via intermolecular proton transfer between the amide units and the fluoride anion, as confirmed by proton nuclear magnetic resonance titration. CA-TBMDS is not only highly sensitive to fluoride anions, but also exhibits high sensitivity in the presence of various ions. This work demonstrates that *N*-butyldimethylchlorosilane-based organic dyes have prospective utility as a type of fluoride anion chemosensor.

## Introduction

Fluoride is among the most electronegative ions and is the smallest anion, with a high charge density. Fluoride plays a key role in human health and chemical engineering because: (i) the fluoride anion is easily absorbed by the animal or human body, but it is excreted slowly. As a result, people or animals develop bone and thyroid activity disorders if they are overexposed to fluoride (Wade et al., 2010). (ii) Fluoride anions play a crucial role in organic synthesis, the chemical industry, biological and medical processes, and the military fields (Kleerekoper, 1998; Cametti and Rissanen, 2009; Wade et al., 2010; Xuan et al., 2013; Zhou et al., 2014; Li et al., 2018). An appropriate amount of fluoride anions in the environment is healthy for humans. However, a large amount of fluoride in the environment is hazardous and even toxic (Kaur and Choi, 2015). With the rapid development of the chemical industry, fluoride anions are present not only in aqueous environments, but also in organic media, such as waste organic liquor (Clark, 1980). The development of highly sensitive and selective fluoride anion sensors capable of qualitative and quantitative detection is crucial and could provide a diversity of optical chemosensors for fluoride anions in organic solutions.

In the past few years, many scientific studies have focused on the development of novel fluoride anion sensors with high sensitivity and selectivity (Yang et al., 2013; Feng et al., 2018; Wang et al., 2018; Antonio et al., 2020). Very recently, aminobenzodifuranone dyes for F^−^ chemosensors were developed by our group, which could not only detect F^−^, but could also distinguish it from F^−^ containing solvents (Deng et al., 2020). Yuan et al. developed a new (3Z, 3′Z)-3,3’-(4,4,9,9-tetrakis (4-hexylphenyl)-4,9-dihydro-s-indaceno [1,2-b:5,6-b']dithiophene)-2,7-diylbis (methan-1-yl-1-ylidene))bis (6-bromo-indolin-2-one) (IDTI) dye with a detection limitation as low as 1 × 10^–7^ M for the fluoride anion (Yuan et al., 2020). Additionally, Zhang and co-workers developed a DPP-based polymer from *t*-butoxy carbonyl (*t*-Boc) units that detect fluoride anions, and also extract fluoride anions from organic solutions (Zhang et al., 2018).

The reported fluoride anion sensors generally react with fluoride anions, resulting in changes in the UV-vis absorption and/or fluorescence emission spectra of the sensors. Among these, the sensors that allow for the detection of color with the naked eye are more interesting and promising because fluoride anions can be detected easily and simply without the need for auxiliary equipment. Herein, a new organic conjugated carbazole small molecule, which can allow for fluoride detection with the naked eye, was developed for use as a fluoride anion chemosensor.

## Materials and Methods

### *N-Tert*-Butyldimethylsilyl-3,6-Diiodocarbazole (CA-TBMDS)

In a dry flask under nitrogen protection, 3,6-diiodocarbazole (2.00 g, 4.77 mmol) was dissolved in anhydrous tetrahydrofuran (THF; 30 ml) at room temperature. Sodium hydride (172 mg, 7.2 mmol) was added to the stirred solution, which was further stirred at room temperature for another 30 min *tert*-Butyldimethylsilyl chloride (0.79 g, 5.3 mmol) was then added and the reaction was stirred at room temperature for a further 17 h. The reaction mixture was then poured into ice water (50 ml) and extracted three times with dichloromethane. The combined organic layers were dried over MgSO_4_ before the solvent was removed *in vacuo*, which afforded an off-white solid. The crude product was purified through a plug of silica in a fitted funnel with hexane/dichloromethane (9:1) as the eluent. After removing the solvent *in vacuo*, the product was obtained (2.06 g, yield: 81%). ^1^H NMR (500 MHz, *d*_*6*_-DMSO) *δ* ppm: 8.61 (s, 2H), 7.65–7.67 (d, J = 10 Hz, 2H), 7.51–7.53 (d, J = 10 Hz, 2H), 0.96 (s, 9H), 0.75(s, 3H).

## Results and Discussion

**FIGURE 1:**
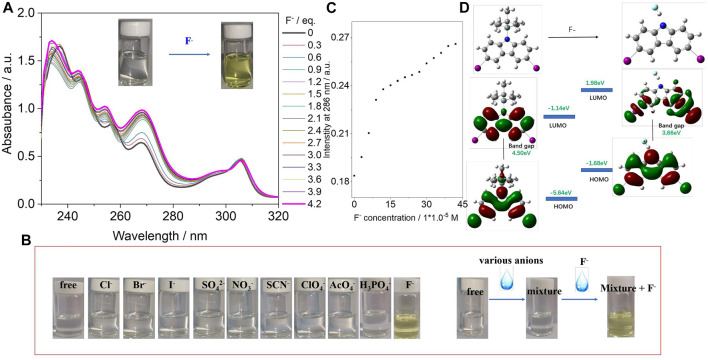
**(A)** UV/vis absorption spectra of CA-TBMDS (1.0 × 10–^4^ M) in the presence of F^−^ (0–4.2 eq.) in THF. Inset shows photograph of CA-TBMDS solution with or without adding different amounts of fluoride anion under ambient light. **(B)** Photographs of CA-TBMDS (5 × 10^−5^ M) in THF before and after adding different anions, as well the addition of other anions together with and without F^−^. **(C)** Optical absorption intensity at 286 nm against the concentration of fluoride anion. **(D)** Molecular orbital surfaces of the HOMO and LUMO energy levels of CA-TBMDS at the B3LYP/6–31G (d, p) level, before and after adding fluoride anion.

**FIGURE 2:**
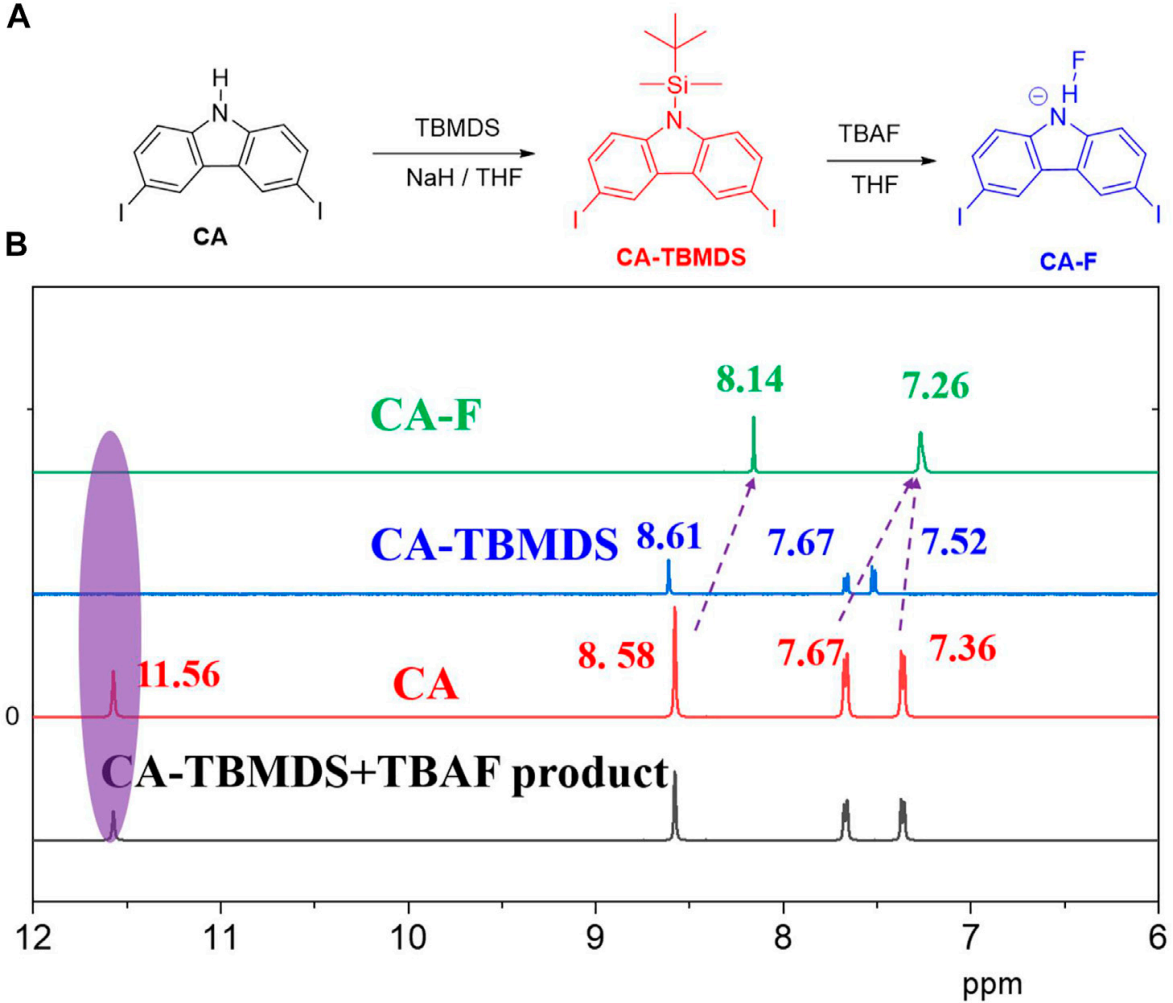
**(A)** Synthesis of CA-TBMDS, and reaction between CA-TBMDS and fluoride anion. **(B)** Partial ^1^H NMR titration spectra of CA and CA-TBMDS before and after adding fluoride anion (4 eq.) in DMSO-*d*_*6*_.

The synthesis of CA-TBMDS is described in the *Materials and Methods* section. CA-TBMDS showed good solubility in most common organic solvents. The interaction between the CA-TBMDS chromophore and fluoride anions was first investigated by using the naked eye to determine the color change. As shown in the inset of Figure 1A, the pure CA-TBMDS solution was colorless. Once the fluoride anion was introduced, the color of the solution immediately changed to yellow. This indicated that in the presence of CA-TBMDS, fluoride anions could be detected by the naked eye without additional equipment. Additionally, CA-TBMDS presented blue emission, which is sensitive to the naked eyes. A spectrophotometric titration was used to investigate the interaction between the CA-TBMDS chromophore and fluoride anion in THF solution. A standard solution of tetrabutylammonium fluoride (TBAF, 1.0 × 10^–2^ M) was gradually added to a 1.0 × 10^–4^ M solution of CA-TBMDS in THF. As shown in Figure 1A, with the progressive addition of fluoride anions, the absorption intensity at 306 nm showed almost no change, but other absorption peaks were slightly blue-shifted, which enhanced the absorption intensity. For example: (i) the absorption peak at 268 nm with an intensity of 0.61 shifted to 267 nm with an intensity of 0.99; (ii) the absorption peak at 256 nm with an intensity of 0.80 shifted to 254 nm with an intensity of 1.01; (iii) the absorption peak at 245 nm with an intensity of 1.27 shifted to 244 nm with an intensity of 1.38; (iv) the absorption peak at 236 nm with an intensity of 1.31 shifted to 234 nm with an intensity of 1.70. In addition, the optical absorption intensity of the spectra vs the concentration of fluoride anion was calculated which was described in Figure 1C. Figure 1C showed that the detection limit of CA-TBMDS for the fluoride anions was at least 3 × 10^–5^ M. As shown in Figure 1B, apart from F^−^ (as tetrabutylammonium salts), anions (4 equivalents) such as Cl^−^, Br^−^, I^−^, SO_4_^2–^, NO_3_^−^, SCN^−^, ClO_4_^−^, AcO_4_^−^, and H_2_PO_4_^−^ caused almost no change in the color of the CA-TBMDS solution. Interestingly, there was no noticeable color change upon the addition of other anions together with F^−^, although F^−^ by itself led to an immediate change from colorless to yellow (Figure 2B). This observation indicates that fluoride anions can be detected without interference from other anions. Thus, CA-TBMDS seems to be a highly sensitive and selective sensor for fluoride anions.

The changes in the color and optical absorption spectrum are plausibly associated with the decomposition of *t*-butyldimethylchlorosilane (TBMDS) from CA-TBMDS. Once CA-TBMDS interacted with the fluoride anion, the CA-TBMDS molecules were transformed to CA, which generated N-H units. This was confirmed by the NMR test. The CA-TBMDS solution was added by TABF solution, subsequently the mixture was stirred for 15 min under room temperature. The mixture was purified by the saturated NH_4_Cl (aq) and extracted with toluene. The combined organic layers were washed with deionized water then dried by MgSO_4_ before the solvent was removed in vacuo to afford the white product. The NMR of the product was measured, which showed the same peaks compared to the CA. Compare to the CA-TBMDS, the peaks of the product were shifted into slightly lower ppm, for instance the proton of the carbazone core of 8.61 ppm shifted into 8.58 ppm, while 7.72 ppm was moved to 7.36 ppm. The lactam NH moiety from the CA core was able to interact with the fluoride anions, which easily deprotonated the -NH protons (inter-molecular proton transfer, IPT, Figure 2A (Zhang et al., 2018)).^1^H NMR experiments were carried out in DMSO-*d*_*6*_ to confirm our assumption and further understand the interaction between the fluoride anion and the CA-TBMDS acceptor. As shown in Figure 2B, except for the signal of the amino proton at 11.56 ppm, the specific signals from the carbazole core of CA and CA-TBMDS were similar. Once the fluoride anions were added, the proton signal of carbazole at 8.58 ppm shifted to 8.14 ppm, while the respective proton signals at 7.67 and 7.36 ppm shifted to 7.26 ppm. This may be due to the IPT process, which severed the TBMDS units from the CA core, and resulted in hydrogen bonding between the fluoride anion and the proton on the amino N-H. In addition, the signals of the amino protons at 11.56 ppm for CA did not appear, which further confirmed this assumption.

To further understand the electron distributions before and after fluoride anion binding, the Frontier molecular orbital (FMO) energy was calculated at the B3LYP/6–31 (d, p) level using CA-TBMDS. As shown in Figure 1D, the distribution of the highest occupied molecular orbital (HOMO) and lowest unoccupied molecular orbital (LUMO) orbitals of CA-TBMDS was similar because these orbitals were mainly localized on the core of CA. After binding with the fluoride anions, the HOMO orbital distributions showed almost no change, but the electron distribution of the LUMO, which was associated with the binding of CA to the fluoride anions, was mainly located at both ends of the CA core. This indicates that when the CA became excited, electron transfer from the core of CA to both ends took place at the fluoride-bonded CA. In addition, after binding with the fluoride anions, the bandgap of the molecules decreased.

## Conclusion

A novel colorimetric chemosensor for detecting fluoride anions was designed and studied. This novel sensor, CA-TBMDS, based on carbazole, exhibits high sensitivity and selectivity for fluoride. CA-TBMDS reacts with fluoride anions in organic solvents, resulting in a visible color change from colorless to yellow, which can be detected with the naked eye. The color change is associated with severance of the TBMDS units from CA-TBMDS by the fluoride anion and simultaneous formation of NH units, leading to intermolecular proton transfer between CA-TBMDS and the fluoride anions. Spectroscopic studies show that for CA-TBMDS, the detection limit for the fluoride anion was as low as 3 × 10^–5^ M. This work demonstrates that CA-TBMDS, with its high sensitivity and selectivity, is a promising dye for fluoride chemosensors, enabling naked eye detection of target analytes. In addition, N-TBMDS units containing organic dyes can be used to produce fluoride anion sensors.

## Data Availability

The original contributions presented in the study are included in the article/Supplementary Material, further inquiries can be directed to the corresponding author.
