# Supplementary figures and images for: Efficient Colorimetric Fluoride Anion Sensor Based on π-Conjugated Carbazole Small Molecule
Source: Front Chem. 2021 Aug 25;9:732935. doi: 10.3389/fchem.2021.732935 (PMC8423907; doi:10.3389/fchem.2021.732935)

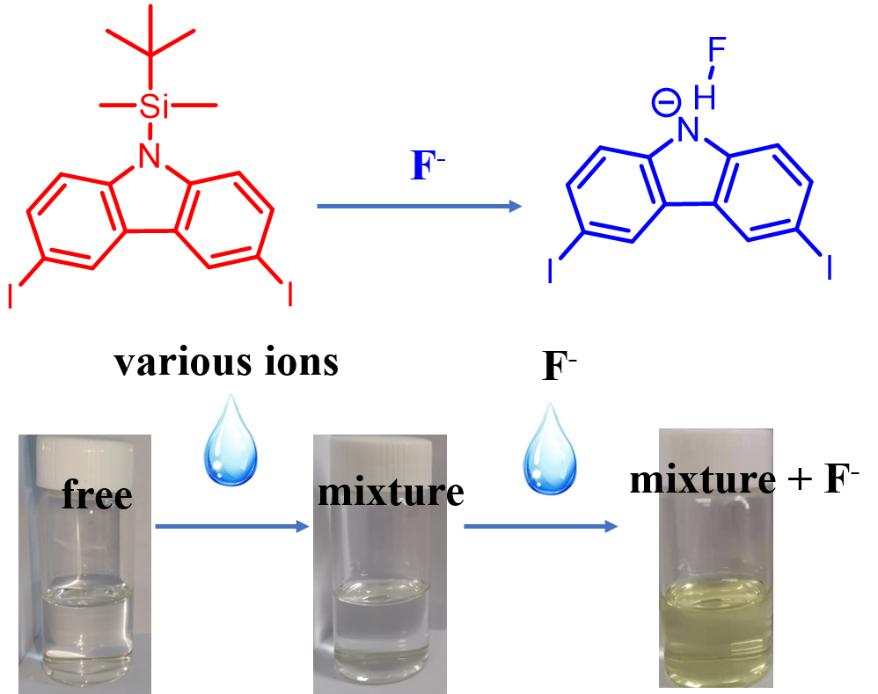

Supplement: Supplementary file 1 [file Image1.JPEG]
